# Supplementary figures and images for: Spatial models of pattern formation during phagocytosis
Source: PLoS Comput Biol. 2022 Oct 3;18(10):e1010092. doi: 10.1371/journal.pcbi.1010092 (PMC9560619; doi:10.1371/journal.pcbi.1010092)

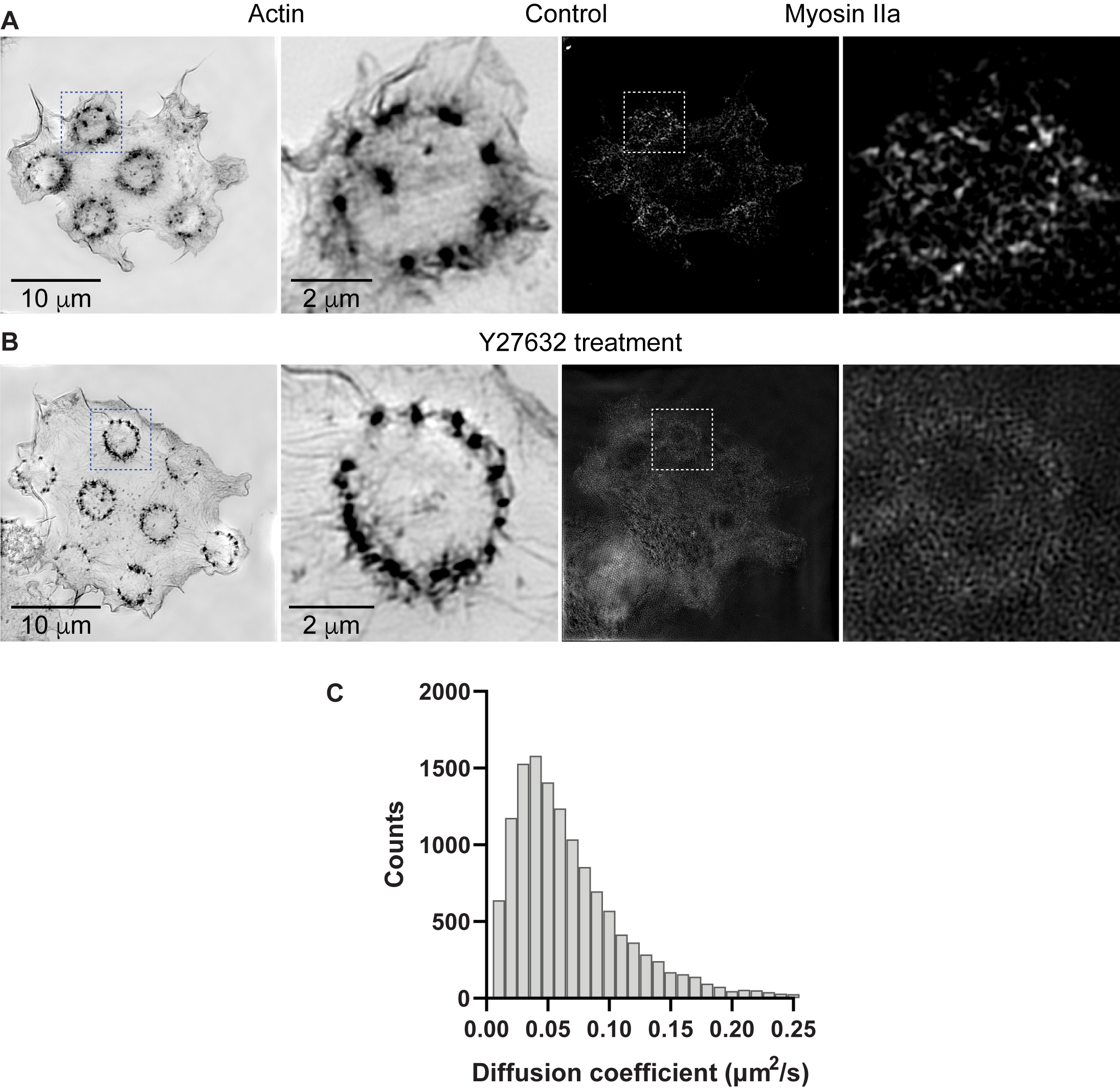

Supplement: S1 Fig — A) Control RAW 264.7 macrophages were marked for actin (phalloidin staining) and myosin II (RLC-eGFP) during frustrated phagocytosis. B) RAW 264.7 macrophages were marked for actin (phalloidin staining) and myosin II (RLC-eGFP) when treated with 20mM Rho kinase inhibitor Y-27632 for 25 min during frustrated phagocytosis. Proper actin rosette formation despite Rho kinase inhibition suggests that actomyosin contractility is not necessary for rosette formation. C) Diffusion coefficient estimates for Cdc42 during frustrated phagocytosis. Quantifications from the mean squared displacement analysis of the single particle tracking performed in Fig 1D-1G. The mode of the distribution is 0.04 μm2 s-1. (TIF) [file pcbi.1010092.s001.tif]

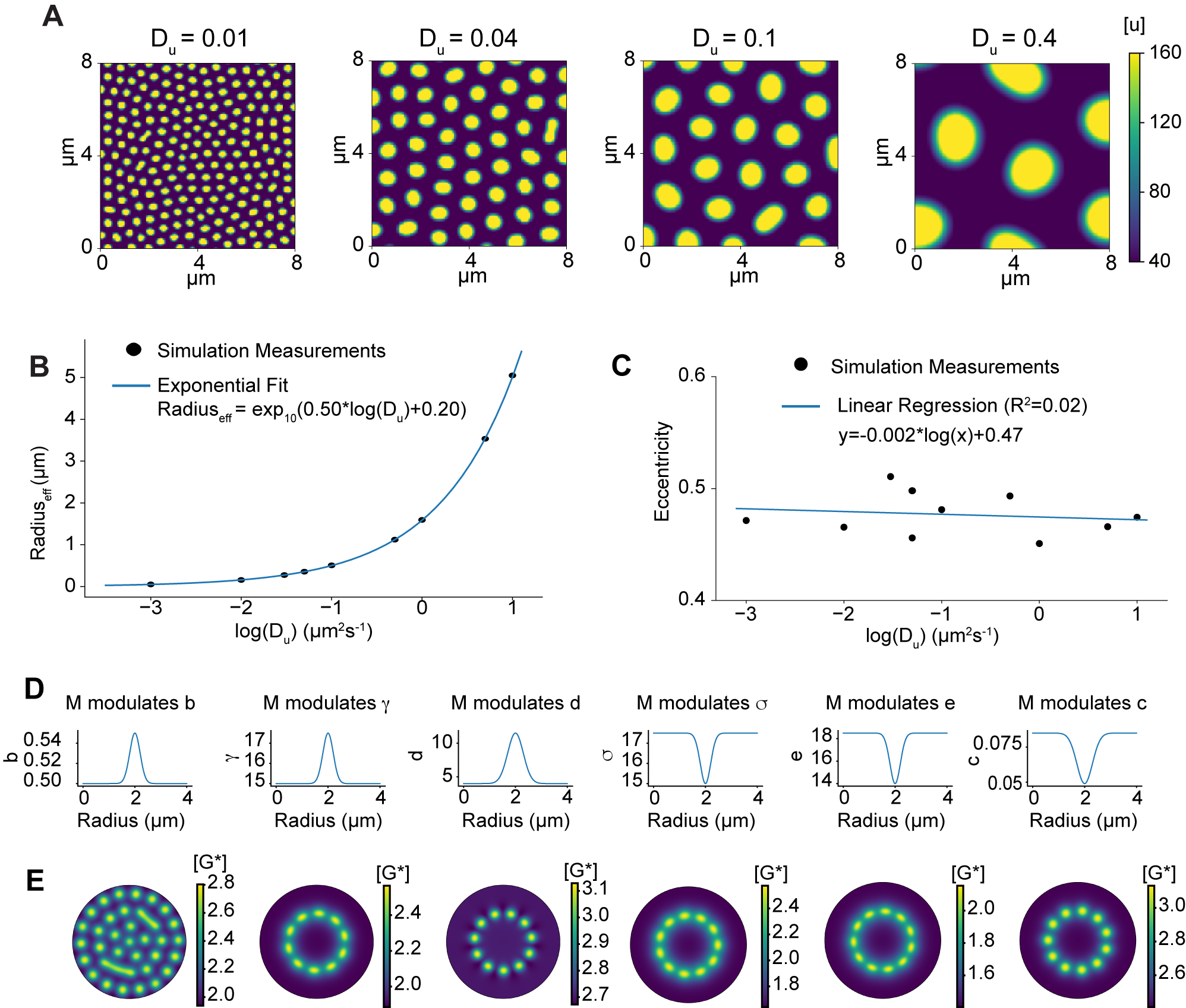

Supplement: S2 Fig — A) Simulations of the WPGAP model for various diffusion rates. The diffusion coefficient for cytosolic species is taken to be 100Du. B) Relationship between the membrane diffusion coefficient and spot size. C) Relationship between the membrane diffusion coefficient and spot eccentricity. D) Radial distributions for the spatial distributions of parameters modulated by an intermediary species M in Fig 2D. E) Active GAP concentrations for the results shown in Fig 2D. (TIF) [file pcbi.1010092.s002.tif]

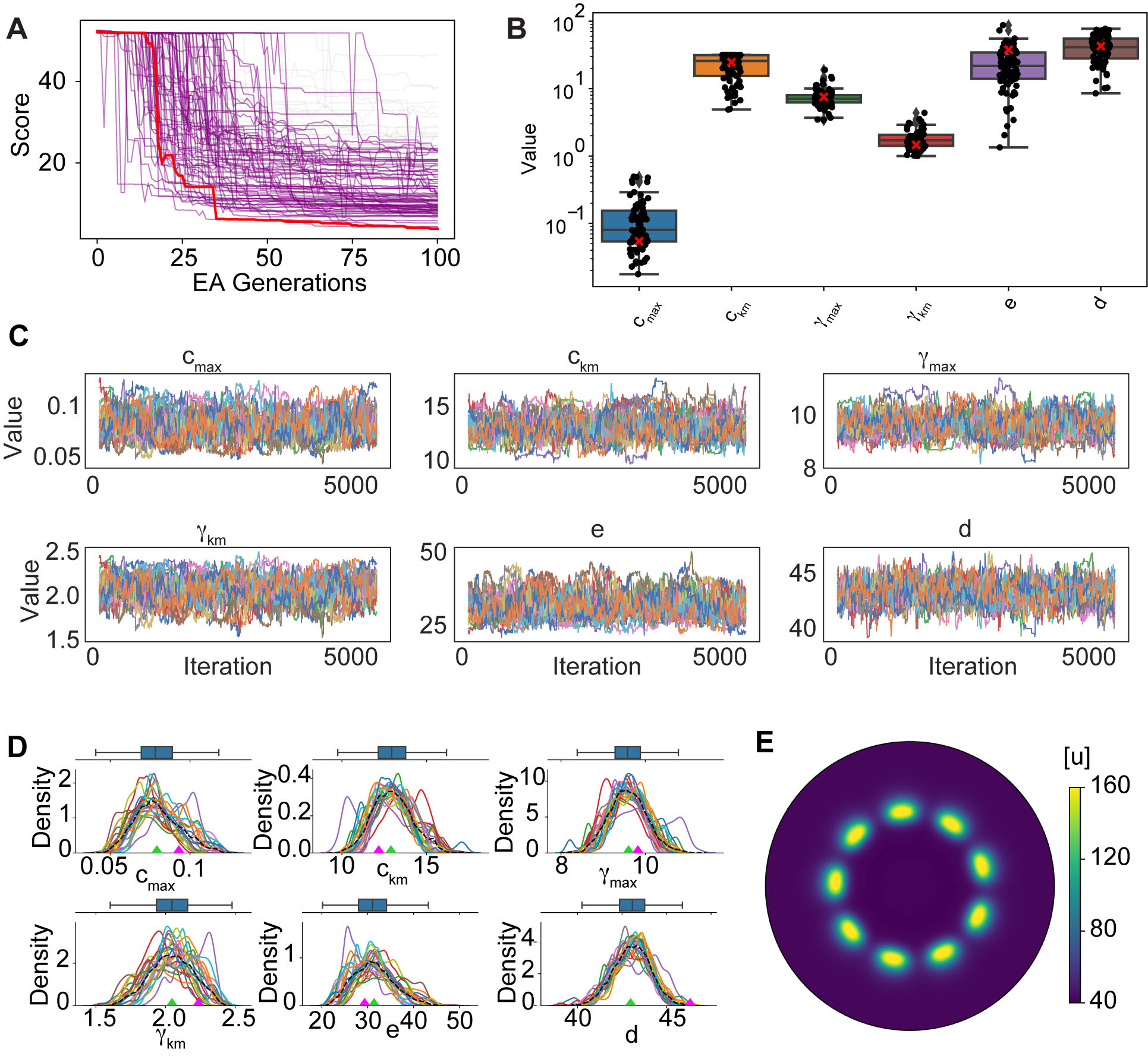

Supplement: S3 Fig — A) EA parametrization runs (99 total). Best individual run shown in red and the runs that resulted in GTPase rosettes shown in purple. B) Individual parameter distributions from the successful EA runs shown in A. The best performing parameter set shown by red crosses. C) DRAM-MCMC chains for individual parameters post burn-in phase. D) Individual parameter densities for the chains shown in C. Representative parameter set values shown by green diamonds (Table 2). The worst scoring parameter set shown by magenta diamonds (S1 Table). E) Active GTPase concentration for the worst scoring parameter set (S1 Table). (TIF) [file pcbi.1010092.s003.tif]

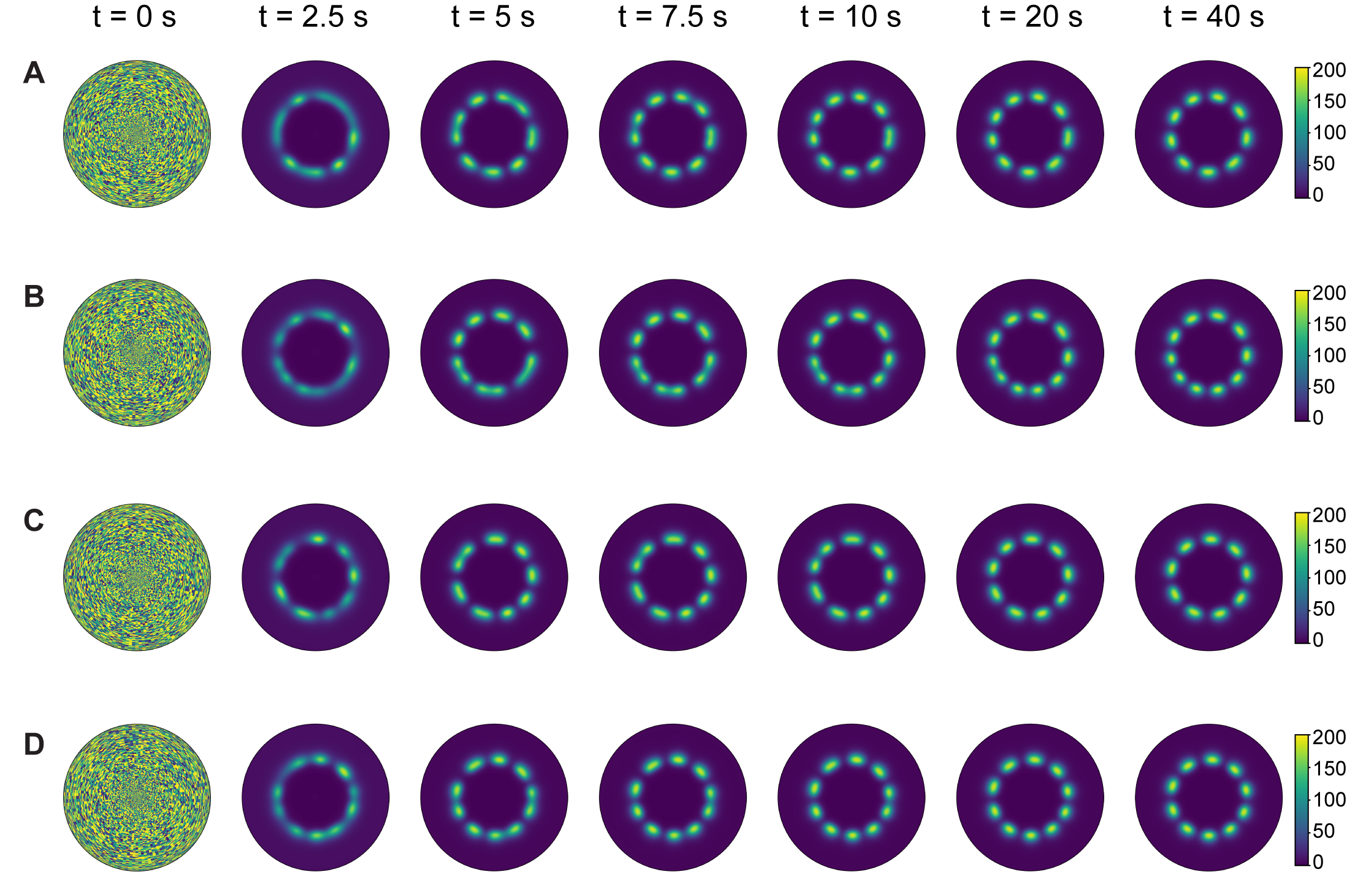

Supplement: S4 Fig — A-D) Four independent simulations of spatially-modulated WPGAP model using the mean parameter values from Fig 4D (on the diagonal, Table 2). Simulations appear to mostly form a rosette within 20 s, with rosettes appearing stable by 40 s. (TIF) [file pcbi.1010092.s004.tif]

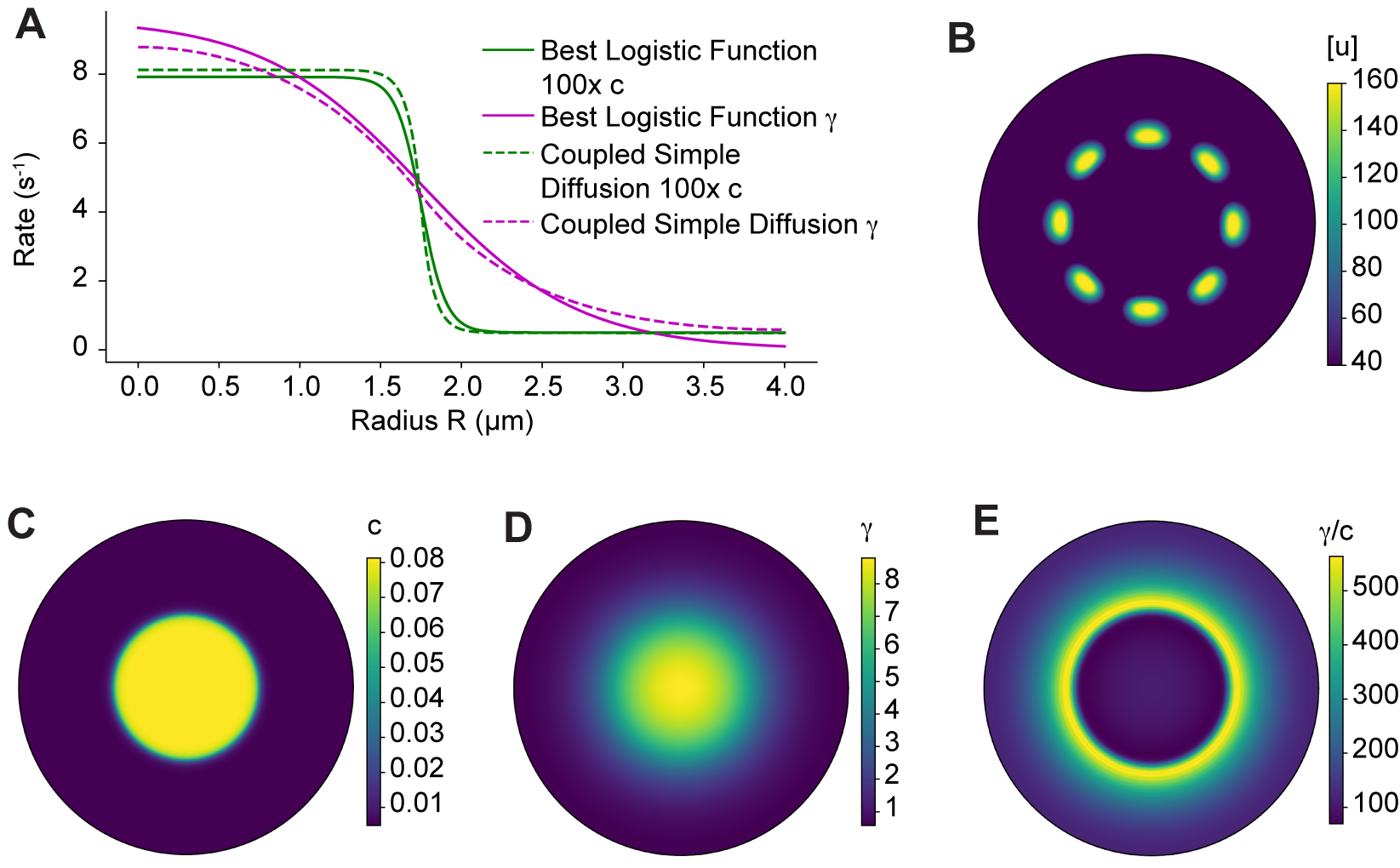

Supplement: S5 Fig — A) Radial distributions for c and γ from a coupled model, where the diffusing species are simulated (dashed lines, see S1 Note) and the logistic function approximations using the representative parameter set (Table 2). B) Active GTPase concentration for the coupled model shown in A. C) Spatial concentration of the diffusing species modulating the GAP activation rate, c, in the coupled model in A,B. D) Spatial concentration of the diffusing species modulating the self-positive feedback rate, γ, the coupled model in A,B. E) The positive to negative feedback ratio γ/c forms a ring. For visualization purposes, the GAP activation rate, c, is multiplied by 100 to be a similar order of magnitude to the self-positive feedback rate γ. (TIF) [file pcbi.1010092.s005.tif]

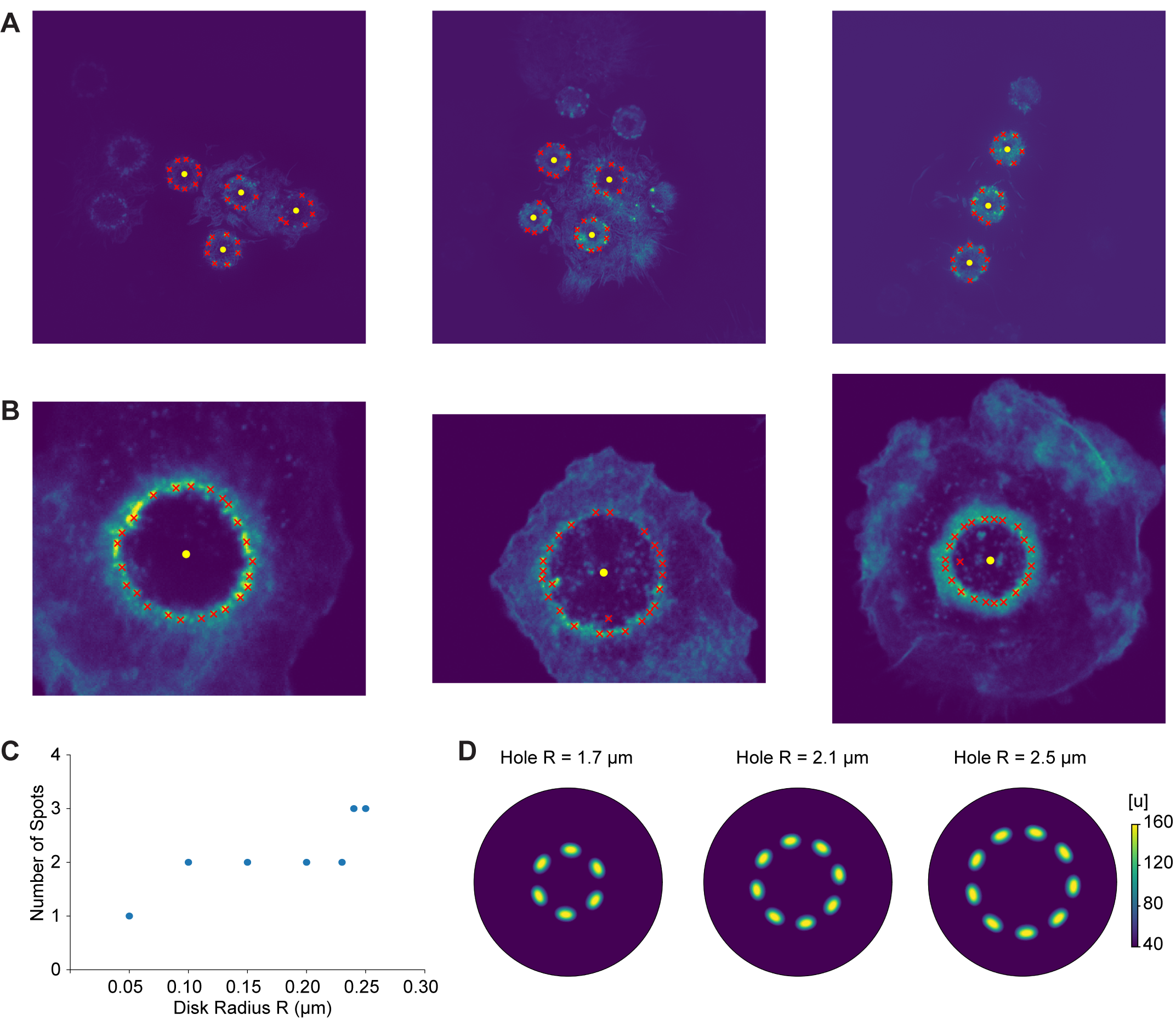

Supplement: S6 Fig — A) Representative experimental results for disks of radius 1.75 μm. Podosomes are indicated with red circles. B) Same as A but using disks of radius 5 μm. C) Simulated number of GTPase spots versus disk radius for small disks with radius less than 0.25 μm. D) Simulations for the representative parameter set (Table 2, negative ckm and γkm) when changing the hole size. (TIF) [file pcbi.1010092.s006.tif]
