# Supplementary material for: Spatial models of pattern formation during phagocytosis
Source: PLoS Comput Biol. 2022 Oct 3;18(10):e1010092. doi: 10.1371/journal.pcbi.1010092 (PMC9560619; doi:10.1371/journal.pcbi.1010092)
Supplement: S1 Table — Fixed values same as in Table 2. (DOCX) [file pcbi.1010092.s007.docx]

| Parameter | Description | Value |
| --- | --- | --- |
| *γ_max_* | GTPase maximal self-positive feedback rate, maximum spatial value | 9.8 s^-1^ |
| *γ_km_* | GTPase maximal self-positive feedback rate, decay rate | 2.24 s^-1^ |
| *c_max_* | GAP activation, maximum spatial value | 0.186 μm^2^s^-1^ |
| *c_km_* | GAP activation, decay rate | 12.2 |
| *d* | GAP inactivation | 46.3 s^-1^ |
| e | GAP dependent GTPase inactivation | 29.3 μm^2^s^-1^ |

**Table S1.** **Worst scoring parameter set after running the MCMC.** Fixed values same as in Table 2.
