# Supplementary material for: Spatial models of pattern formation during phagocytosis
Source: PLoS Comput Biol. 2022 Oct 3;18(10):e1010092. doi: 10.1371/journal.pcbi.1010092 (PMC9560619; doi:10.1371/journal.pcbi.1010092)
Supplement: S1 Note — Details for using reaction-diffusion equations to determine spatially-dependent rates rather than the logistic function approximation. (DOCX) [file pcbi.1010092.s008.docx]

**S1 Note**

In the main manuscript, the profiles of the spatially dependent rates *γ(r)* and *c(r)* were prescribed using the logistic function *f(r)*. Therefore, we wanted to confirm that when we use the solution to the equation for a diffusing species X that is activated over the disk to generate spatial profiles for the chemical species that modulate *γ(r)* and *c(r)*, the system still generates a rosette pattern. As noted in the main text the equation for X is:

$$\frac{\partial X}{\partial t}=k_{x}\left( r \right)-\delta_{x}X+\frac{D_{x}}{r}\frac{\partial}{\partial r}(r\frac{\partial}{\partial r})X$$

$$k_{x}\left( r \right)=\left\{ \begin{aligned} k_{disk} \mathrm{if} r< \mu_{F} , \\ k_{basal} \mathrm{if} r\geq\mu_{F} , \end{aligned} \right.$$

where *k_x_(r)* is the spatially dependent activation rate, *δ_x_* is the deactivation rate and *D_x_* is the diffusion coefficient for X, *k_disk_* is the IgG-induced activation rate, *k_basal_* is the basal activation rate, and *μ_F_* is the radius of the disk.

Surprisingly, however, fitting solutions for X to *γ(r)* and *c(r)* from the main text (logistic function approximation) and using these solutions in the WPGAP model did not lead to proper rosette formation. This discrepancy likely arises from slight differences between the logistic function and solutions to the reaction-diffusion equation (Fig 3D). Therefore, we used the parameter values found from fitting *γ(r)* and *c(r)* to initialize another DRAM-MCMC run that included equations for species that modulate *γ(r)* and *c(r)*. Because our goal was to simply demonstrate proof of principle, we only performed 22 short (1000 iterations) DRAM-MCMC runs, and we took the single best scoring parameter set. However, this was sufficient to demonstrate that a model that explicitly considered species that modulate *γ(r)* and *c(r)* was able to generate a GTPase rosette (S5 Fig, S1 Note Table A, below). In S5C,D Fig, we plot the distributions of the species modulating the rates for the GAP activation rate, *c*, and the self-positive feedback rate, *γ*, respectively. Interestingly, if these profiles are used to modulate the intermediate species M in the two-step model, the system creates a ring of active M (as in S5E Fig). Thus, the same mechanism where the positive feedback strength is lower, but transitions less rapidly than the negative feedback strength can also be used to create an initial ring which could then drive rosette formation. Finally, we note that with the right choice of parameter values, this model can also generate a ring, which could then be used to drive rosette formation (examples of this ring formation can be observed in Fig 5A-F).

| Parameter | Description | Value |
| --- | --- | --- |
| *k_disk - c_* | On disk activation rate of a species modulating *c* | 113 s^-1^ |
| *k_basal - c_* | Off disk/basal activation rate of a species modulating *c* | 6.8 s^-1^ |
| *δ_c_* | Inactivation rate of a species modulating *c* | 6.96 s^-1^ |
| *D_c_* | Diffusion coefficient of a species modulating *c* | 0.04 μm^2^s^-1^ |
| *k_disk − γ_* | On disk activation rate of a species modulating *γ* | 63.3 s^-1^ |
| *k_basal − γ_* | Off disk/basal activation rate of a species modulating *γ* | 9.9e-5 s^-1^ |
| *δ_γ_* | Inactivation rate of a species modulating *γ* | 5.06 s^-1^ |
| *D_γ_* | Diffusion coefficient of a species modulating *γ* | 4.21 μm^2^s^-1^ |

**S1 Note Table A.** **Parameters for describing diffusing species that modulate the GAP activation rate, *c*, and the self-positive feedback rate, *γ*.**
